# Dense monocular depth estimation for stereoscopic vision based on pyramid transformer and multi-scale feature fusion

**DOI:** 10.1038/s41598-024-57908-z

**Published:** 2024-03-25

**Authors:** Zhongyi Xia, Tianzhao Wu, Zhuoyan Wang, Man Zhou, Boqi Wu, C. Y. Chan, Ling Bing Kong

**Affiliations:** 1 College of New Materials and New Energies, Shenzhen Technology University, Shenzhen, 518118 Guangdong China; 2 College of Applied Technology, Shenzhen University, Shenzhen, 518000 Guangdong China; 3 Jilin Jianzhu University, Changchun, 130118 Jilin China

**Keywords:** Depth estimation, Transformer, Deep learning, Stereoscopic display, SPT-depth, Loss function, Engineering, Mathematics and computing, Computational science, Computer science

## Abstract

Stereoscopic display technology plays a significant role in industries, such as film, television and autonomous driving. The accuracy of depth estimation is crucial for achieving high-quality and realistic stereoscopic display effects. In addressing the inherent challenges of applying Transformers to depth estimation, the Stereoscopic Pyramid Transformer-Depth (SPT-Depth) is introduced. This method utilizes stepwise downsampling to acquire both shallow and deep semantic information, which are subsequently fused. The training process is divided into fine and coarse convergence stages, employing distinct training strategies and hyperparameters, resulting in a substantial reduction in both training and validation losses. In the training strategy, a shift and scale-invariant mean square error function is employed to compensate for the lack of translational invariance in the Transformers. Additionally, an edge-smoothing function is applied to reduce noise in the depth map, enhancing the model's robustness. The SPT-Depth achieves a global receptive field while effectively reducing time complexity. In comparison with the baseline method, with the New York University Depth V2 (NYU Depth V2) dataset, there is a 10% reduction in Absolute Relative Error (Abs Rel) and a 36% decrease in Root Mean Square Error (RMSE). When compared with the state-of-the-art methods, there is a 17% reduction in RMSE.

## Introduction

Depth estimation is a fundamental problem in computer vision, allowing users to objectively perceive realistic scenes from flat images^1^. It is crucial in many scenarios, including robot navigation, 3D reconstruction, virtual reality and autonomous driving^2^. Specifically, monocular depth estimation is aimed to extract high-quality scene depth information from individual images and predict the depth values for each pixel in a given image.

Currently, research in monocular depth estimation primarily revolves around two aspects: the architecture of monocular depth estimation networks and the optimization of monocular depth estimation algorithms. Through the optimization of deep learning algorithms, the accuracy of depth estimation has been significantly improved. Convolutional Neural Networks (CNNs)^3^ have emerged as the primary tools in the research of depth estimation algorithms^4–7^. These models employ pixel-wise learning techniques to convert RGB (RGB stands for red, green, and blue, the three primary colors of light used in additive color models.) data into depth maps. However, as the volume of data increases, CNNs have not shown the corresponding adaptability. Challenges persist in CNNs technologies, including low accuracy in predicting depth maps and insufficient clarity in structural features.

From CNNs to Transformers, significant advancements have been made in deep learning. Transformers primarily employ unsupervised learning, whereas CNNs predominantly rely on supervised learning. When designing model architectures, training strategies should be selected based on specific contexts. In automatic stereoscopic display, achieving sufficiently high generalization performance is required to ensure robustness performance and broad applicability of the model. However, the accuracy of depth values plays a critical role in enhancing the final presentation quality of stereoscopic display. Consequently, this paper adopts a supervised training approach is adopted to improve the accuracy of depth estimation, thereby enhancing the overall effectiveness of the stereoscopic display system. In the field of computer vision, CNNs are frequently employed. They leverage local context information, weight sharing, and translation invariance in the convolution process, significantly enhancing the performance of neural networks^8^. CNNs have become the primary technology in computer vision domain. However, it is worth noting that, in most CNNs, 3 × 3 convolution processes are used, which limit the networks' perceptual range. In complex prediction tasks, such as object recognition, depth estimation and semantic segmentation, acquiring global information is essential for maintaining model consistency. To obtain more accurate relative depth information in monocular depth estimation, global information can be utilized to smooth the differences in input feature maps. It is well known that, by stacking multiple convolutional layers, perceptual range of CNNs can be expanded, which is crucial for capturing global context information. The encoder-decoder^9,10^ architecture with stacked convolutional layers is one of the most popular approaches in monocular depth estimation^11^.

The first supervised learning method for monocular image depth estimation was reported by Eigen et al^12^. In current techniques, images are typically sent to the network for convolutional processing. Nevertheless, the convolution process is confined to local information in the input images and cannot establish remote connections between images. This limitation in information affects the accuracy of depth prediction in practical scenarios^13^. To model long-distance dependencies, a self-attention mechanism was initially developed to calculate sequence^14^ representations by connecting different positions in a sequence. In the field of computer vision, the development of similar tasks has been driven by the self-attention mechanism of Transformers and its competitive modeling capabilities. The first pure Transformer design applied directly to a series of image patches for classification tasks was introduced by Dosovitskiy et al. as the Vision Transformer (ViT)^15^. Several upgraded versions of Transformers have been reported, including the Stereo Transformer (STTR)^16^ and Dense Prediction Transformer (DPT)^17^, which have achieved outstanding performance in computer vision tasks with high computational costs when compared to ViT.

The success of Transformer in the domain of computer vision owes to several pivotal factors. Firstly, the attention mechanism present in ViT enables them to learn global dependencies within images, thereby acquiring more comprehensive feature representations. This capability is particularly crucial in tasks such as depth estimation, where a thorough understanding of the overall image structure is paramount. Secondly, ViT exhibits seamless scalability to larger image sizes, which is vital for handling high-resolution imagery. Additionally, ViT demonstrates strong robustness against image noise and occlusion, enabling effective processing of low-quality images. Lastly, the parallel processing capability of ViT contributes to its seamless integration into large-scale image processing tasks. Despite the aforementioned advantages of ViT, they still face several challenges, including high computational costs, lower precision in capturing fine-grained edge details, and excessive parameter scales. Addressing these challenges is crucial for the broader adoption of ViT in real-world applications.

Addressing the computational cost issue of ViT, researchers have begun exploring solutions that combine CNNs with ViT, aiming to reduce computational costs and parameter sizes while retaining a global receptive field. The Swin Transformer, developed by Liu et al^18^., combines the advantages of both CNNs and Transformer, restricting attention costs within a window, significantly reducing computational complexity while maintaining efficiency by decreasing computational time complexity. Similarly, the Self-Attention-Based Visual Depth Network (SABV)^19^, developed by Wang et al., enhances monocular depth estimation by integrating a self-attention mechanism inspired by the biological visual system, thereby improving prediction accuracy and presenting richer object details in depth maps. Recently, self-supervised monocular depth estimation has attracted widespread attention, with a focus on designing lightweight yet effective models for deployment on edge devices. Zhang et al^20^. proposed a lightweight monocular depth estimation hybrid architecture, achieving comparable results by efficiently combining CNNs and Transformers, demonstrating state-of-the-art performance on datasets such as Karlsruhe Institute of Technology and Toyota Technological Institute (KITTI). Yang et al^21^. proposed a simple yet powerful monocular depth estimation base model, named Depth Anything. They employed a data engine to collect and automatically annotate images, significantly expanding the data coverage. Additionally, they utilized data augmentation techniques to create more challenging optimization targets and employed pre-trained encoders to acquire semantic priors, thereby enhancing robustness.

Based on the latest developments in monocular depth estimation frameworks, current techniques mainly exhibit the following shortcomings: (1) Transformers excel in capturing crucial global information for depth estimation but lack translation-invariant features present in CNNs. This limitation becomes evident when applying Transformers to depth estimation, as it results in the model lacking spatial information during depth estimation. (2) ViT models typically have a large number of parameters, making them resource-intensive during both training and inference. This might be impractical for many edge devices and resource-constrained environments. Furthermore, compared with traditional convolutional operations, self-attention mechanisms may lack an understanding of spatial relationships between local pixels when processing images. This could lead to inferior performance in predicting texture information, such as edge detection, as compared with CNNs.

Therefore, we propose SPT-Depth to address these issues. We use Scale and Shift Invariant (SSI)^22^ loss to tackle the lack of translation invariance in Transformers. By introducing a Pyramid Transformer as a backbone, we reduce the dimension of feature maps through downsampling to decrease the number of tokens, thereby reducing computational load. The method proposed in this paper achieves very competitive results in terms of RMSE and Abs Rel.There are two variants of SPT: SPT-base and SPT-large.

## Related works

CNNs find extensive applications in computer vision. The convolutional operation, incorporating local context information, weight sharing, and translation invariance, significantly enhances the effectiveness of neural networks. To extract useful information from multi-scale maps and generate multi-scale feature maps, Disparity Network (DispNet) based on U-Net, proposed by Ronneberger et al^23^., is a typical encoder-decoder architecture. Godard et al^7^. introduced a Residual Network-based (ResNet)^24^feature encoder Monocular Depth Estimation 2, making it a widely adopted standard approach. To bridge the semantic gap between the encoder and decoder in deep networks, Lyu et al^25^. redesigned skip connections in the U-Net architecture by fusing features from different scales. In this mode, the performance of depth estimation, semantic segmentation, and instance segmentation models on each task outperforms competitors trained separately. Peng et al.^26^ generated the best depth map from the multi-scale outputs of the network and used this extracted depth map to train the same network. While this approach can improve model accuracy, we believe it partly offsets the advantages of self-supervised mode. Moreover, the additional constraint information significantly increases the number of model parameters.

The original purpose of the Transformer was to capture long-range dependencies in textual information, but it quickly found applications in the field of computer vision. Various self-attention networks have demonstrated significant advantages over mainstream CNNs in various visual tasks. ViT has also been extended to address dense prediction problems, such as depth estimation. For instance, Detection Transformer (DETR)^27^ is the pioneering model utilizing transformers for dense prediction tasks. DETR divides the input image into multiple patches and merges them. Global Filter Network (GFNet), proposed by Rao et al^28^., optimizes spatial connectivity weights in the Fourier domain, equivalent to circular global convolution in spatial dimensions. Wang et al^29^. designed a Pyramid Vision Transformer (PVT) suitable for dense prediction tasks. It overcomes the challenges of porting Transformers to various dense prediction tasks, making it a unified backbone for various visual tasks. Wang et al^19^., inspired by biological visual interaction mechanisms, improved information retention capacity by focusing on information transfer between each module of the network, enabling the network to output depth maps with rich object information and detail. By studying the interpretable relationship between the biological visual system and the monocular depth estimation network, it concretizes the attention mechanism in biological vision. Finally, Zheng et al^30^. designed a new framework, A hybrid of a Convolution, self-attention, and an Multilayer Perceptron (MLP)^31^ network (CSMHNet), by combining decomposed large kernel convolutions and multi-layer perceptron to overcome the shortcomings of convolutional static weights and locality, while significantly reducing memory overhead compared to the Transformer architecture.

CNNs can generally be divided into different blocks, and at the beginning of each block, the length and width of the feature map are halved, while the feature dimension (channel) is doubled. There are two main considerations for this: one is that using convolutional or pooling layers with step size 2 for feature dimension reduction can increase the receptive field and reduce computation, while compensating for spatial loss with an increase in channel dimension. In comparison, ViT has a global receptive field, so ViT can directly tokenize input images and continuously stack the same Transformer Encoder layers, which is feasible for image classification. However, when applied to dense tasks, it encounters the following problems: First, semantic segmentation and depth estimation often require higher resolution inputs, and when the input image size increases, the computational cost of ViT increases sharply; Second, ViT directly uses larger patches for tokenization, such as patch size of 16, resulting in coarse-grained features that incur significant loss for dense tasks. Using PVT as the backbone network does not encounter the above-mentioned problems. PVT adopts a hybrid pyramid architecture of Transformer, dividing the network into different stages, and each stage reduces the H and W of the feature map by half compared to the previous one, meaning that the number of tokens is reduced by 4 times. At the same time, to further reduce computation, PVT replaces the conventional multi-head attention (MHA)^13^ with Spatial Reduction Attention (SRA)^29^. The core of SRA is to reduce the number of key and value pairs in the attention layer. In the conventional MHA, the number of key and value pairs in the attention layer calculation is the length of the sequence, but SRA reduces it to 1/R2 of the original length. In terms of specific accuracy, although PVT does not significantly improve over ViT in terms of accuracy, it can significantly reduce computation and output multi-scale feature maps, which is crucial for segmentation and detection. Most segmentation and detection models currently use the Feature Pyramid Network (FPN) structure, and the feature of PVT can seamlessly serve as a replacement for the backbone of CNNs, connecting segmentation and detection heads.

Taking into account the above improvements to the monocular depth framework, we have made a series of improvements to the model itself. In this paper, we construct a novel encoder-decoder architecture that combines the strengths of Transformer and CNNs. Using PVT as the backbone for dense prediction tasks, it extracts multi-scale feature maps, and through a series of operations including embedding, upsampling, resampling, and convolution, it restores multi-scale feature maps to the same resolution and fuses multi-level information to complete the depth estimation task.

## Method

**Figure 1:**
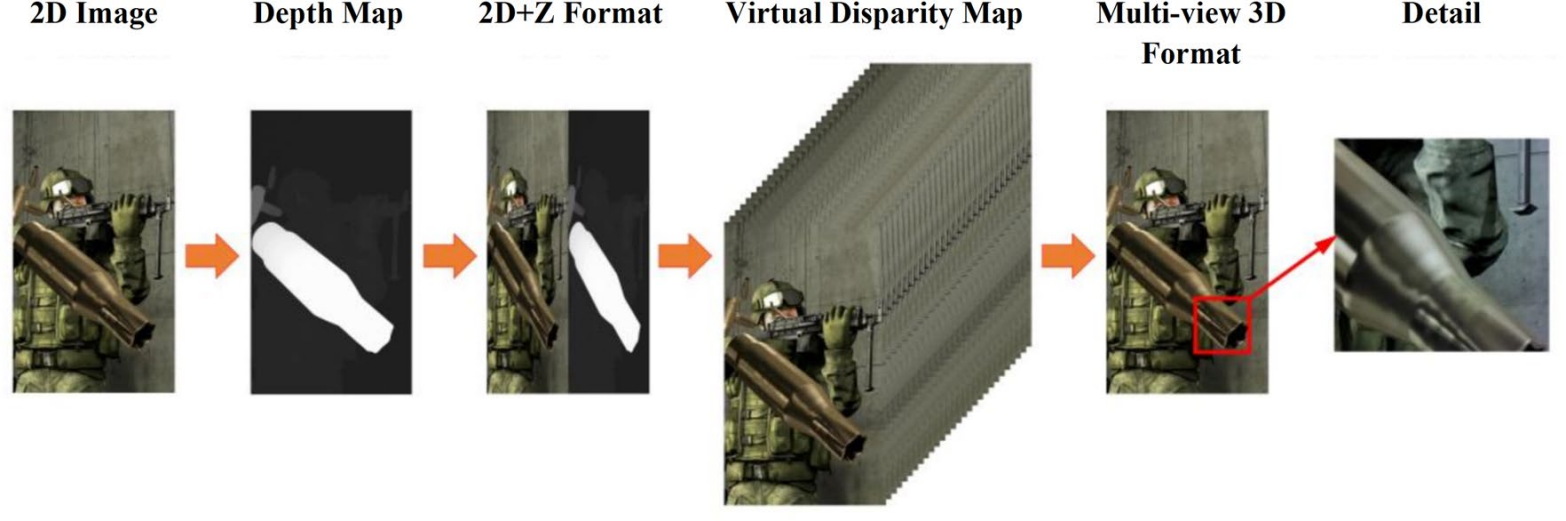
Process from 2D image to stereo vision.

Figure 1 illustrates the process of transforming a 2D image into Multi-view 3D Format in automatic stereoscopic display devices, with the specific steps outlined as follows: (1) Convert the input 2D image into a depth map through depth estimation. This is also a crucial step, as the accuracy and quality of the depth map directly determine the effectiveness of subsequent stereoscopic display. (2) Stitch the images into a 2D + Z format. (3) Use Depth-Image-Based Rendering (DIBR)^32^ techniques to render the 2D + Z format image, synthesizing content from the three-dimensional scene into the two-dimensional image. (4) Synthesize multi-view^33^ images to display the final stereoscopic effect on the screen.

**Figure 2:**
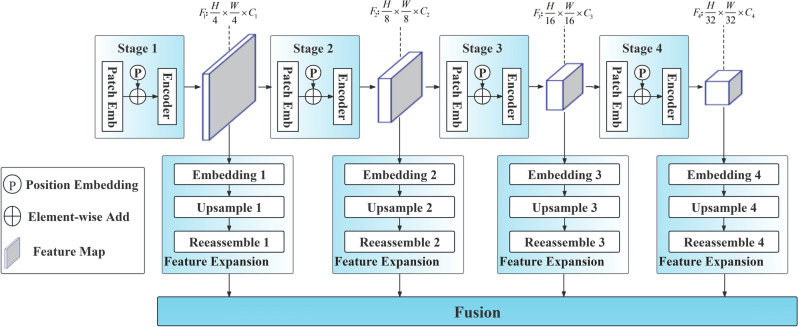
Architecture overview. Before entering the encoder, the image undergoes several steps. Firstly, feature extraction is performed using patch embedding to convert the feature map into tokens (2D), along with relative positional encoding to preserve spatial information. Then, the encoder extracts high-level abstract features such as edges, textures, and shapes. Finally, there is a reshaping step to restore the tokens into a three-dimensional feature map. This process is repeated four times to achieve a pyramid-style down-sampling. Since each feature map has different resolutions and multi-scale information, the feature maps go through an "Expansion" process to restore them to the same resolution size for feature fusion. It is noteworthy that in our training process, we have frozen a significant portion of the weights in the PVT encoder. To enhance the performance of the backbone network in depth estimation, we employ fine-tuning strategies within the MHA module, such as incorporating low-rank trainable matrices into the Linear layers. Subsequently, the feature maps (tensors) generated through progressive downsampling are utilized for decoding. We have drawn inspiration from ResNet to accomplish multi-scale feature fusion. Before feeding the feature maps into the decoder, they undergo another round of embedding. The reason is evident: feature fusion requires tokens (2D), while the up-sampled feature maps (3D) do not align with this requirement.

The SPT-Depth network leverages an encoder-decoder architecture^6^. In Fig. 2, the role of the PVT encoder is to generate high-quality features as the starting point for model training. These features, which integrate shallow and deep semantic information, are reshaped into tokens and then restored to the same resolution using different magnification upsampling schemes for feature fusion.

**Figure 3:**
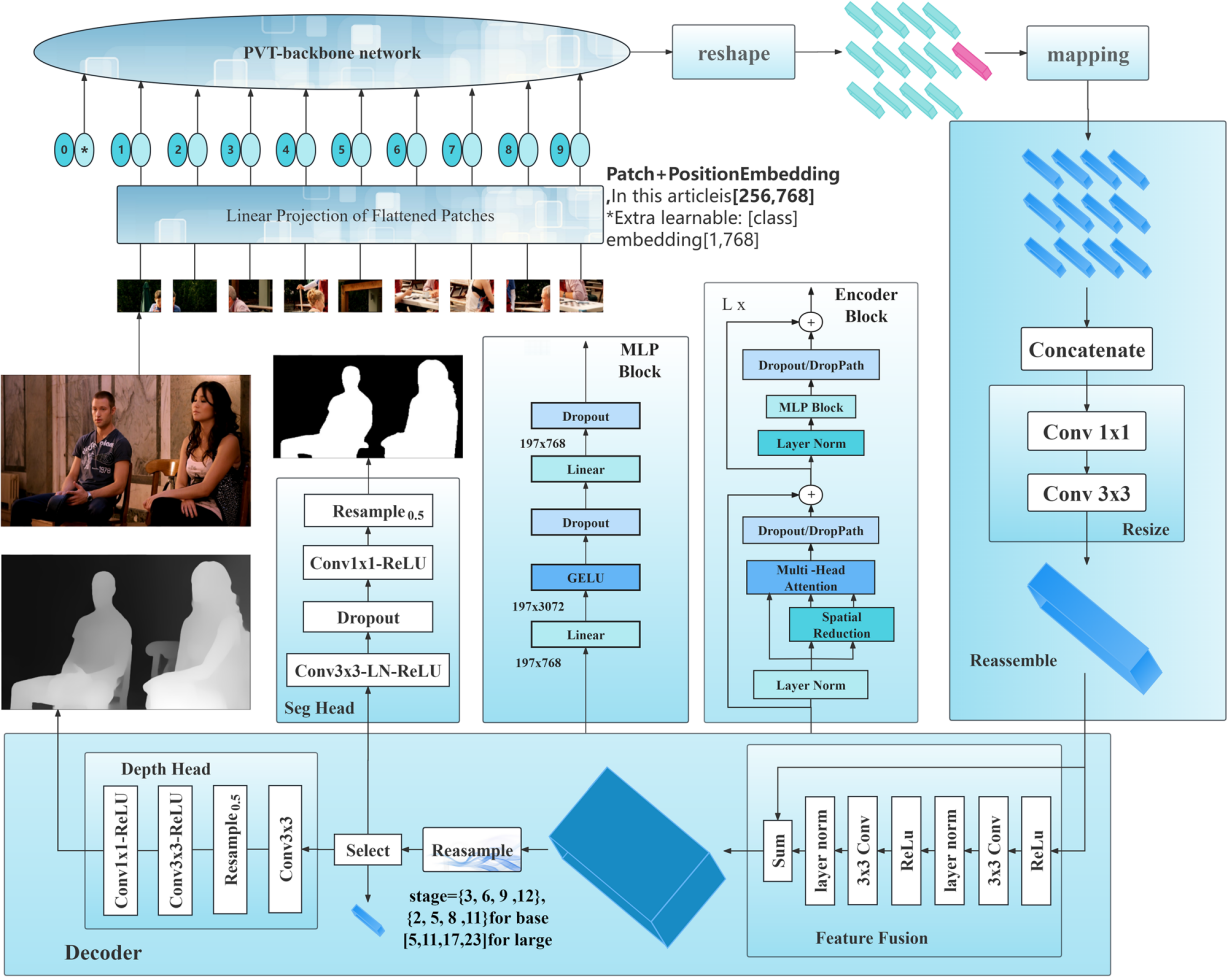
Implementation Details. Firstly, the encoder generates four feature maps F1-F4 through progressive downsampling, which are then transformed into tokens under reshaping. Subsequently, under the mapping process, these tokens are mapped to high dimensions, and then restored to three-dimensional features through concatenation for convenient resizing by subsequent convolutions. Under resampling, these feature maps are upsampled to the same shape while preserving shallow and deep semantic information. Finally, multi-scale feature fusion is accomplished through fusion. In the encoder, we employ Linear SRA, which reduces time complexity through Spatial Reduction. On top of frozen Linear SRA weights, we use trainable weight matrices with ranks lower than the original SRA to fine-tune the encoder (for example, for a tensor shape of (196,768), it would be decomposed into two trainable weight matrices of sizes 1961 and 1768, preserving the original weights in a similar manner to ResNet). In the MLP, we replace the activation function with Gaussian Error Linear Unit (GELU) to enhance the model's robustness to noise and data biases.

The implementation details of SPT are illustrated in Fig. 3. In the experiments, we conducted two reshaping operations: one before mapping to convert features into tokens for high-dimensional mapping, and another during concatenation to reshape tokens into features for convolutional operations. This is because, in the fusion stage, besides considering the use of bilinear interpolation for upsampling and residual connections for fusing multi-scale features, using low-rank fine-tuning attention for fusion is also one of the approaches. However, this method should be used with caution because using attention on small datasets is more prone to overfitting, and trainable parameters composed of low-rank matrices may impact the model's robustness, despite its significant potential for improving model performance.

### Encoder

#### Embedding

The encoder requires the input to be a sequence of tokens (vectors), characterized as a two-dimensional matrix [num_token, token_dim]. Since the format of 3D matrix for image data is [H, W, C], it is obvious that this 3D matrix is not the required one by the encoder, so Embedding is used to transform the data to meet the encoding requirements. Firstly, the images are divided into non-overlapping blocks (num_token) with a given patch. Secondly, each num_token is mapped into a one-dimensional vector by linear mapping. Finally, the mapping dimension token_dim is permuted using patch to obtain a two-dimensional matrix [num_token, token_dim] of the input token (vector) sequence suitable for encoding.

This principle is implemented with a 16 × 16 convolution, 768 channels, and stride 16, transforming the input feature map from [224, 224, 3] to [14, 14, 768]. The output is reshaped to a [196, 768] matrix.

The approach of PVT involves adding a [class] token in Stage 4, which is a trainable parameter and represents a vector of size [1, 768]. This [class] token is then concatenated with the rest of the tokens, forming a two-dimensional matrix of size [197, 768], which is subsequently fed into the encoder. At this point, its shape matches that of the positional embeddings, which is [197, 768].

#### MHA and linear SRA

SRA^29^ is built upon the MHA^13^, aiming to further reduce computational complexity by decreasing the number of key-value pairs in the attention layer. In conventional MHA, the number of key-value pairs is equivalent to the length of the sequence; however, SRA divides the feature map into patches, linearly transforming patches into $\frac{HW}{{R^{2} }} \times C$, thereby reducing the number of key-value pairs to $\frac{1}{{R^{2} }}$ of the original count. Linear SRA^34^ is an improved version of SRA. It achieves resolution reduction by replacing convolutional operations with a combination of pooling and convolution operations. Prior to the attention operation, an average pooling is applied to reduce the spatial dimensions $(h \times w)$ to a fixed size $(P \times P)$, where $P$ represents the pooling size in Linear SRA^34^. Therefore, Linear SRA incurs linear computations and memory overhead, similar to convolutional layers. Since Transformers can only represent sequence lengths L, while in Vision Transformers, images are represented in two dimensions, it is necessary to convert the sequence into the length and width of an image to describe the time complexity of different attention methods. Replacing $L$ with $h \times w$, we obtain $\Omega (Linear\;SRA) = 2hwP^{2} c$.

**Figure 4:**
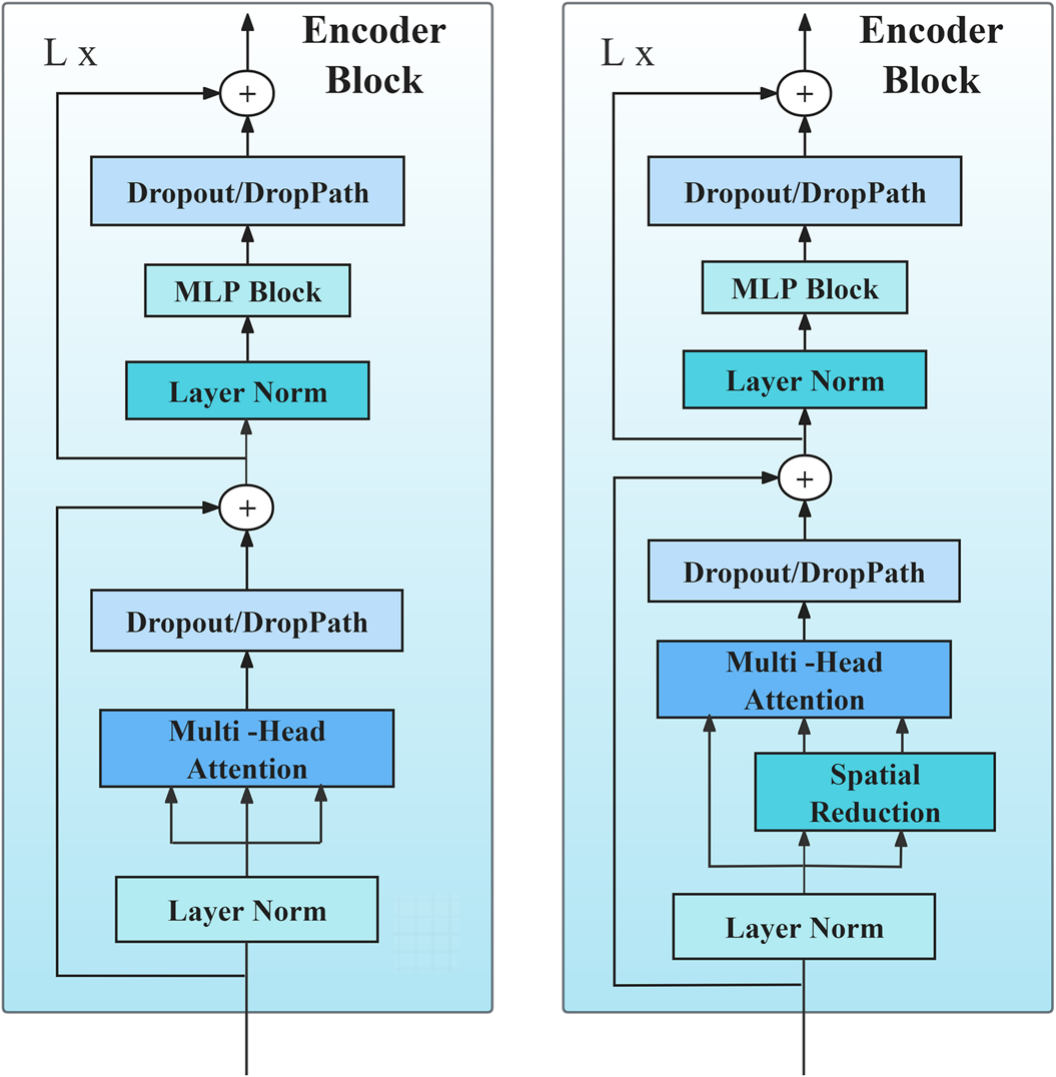
MHA and Linear SRA.

Both Linear SRA and MHA can focus on inputs in different semantic spatial dimensions by dividing the hidden state vectors into multiple heads, generating numerous sub-semantic spaces. As shown in Fig. 4, Spatial Reduction reduces the resolution of the feature maps of the MHA's input Key and Value from the original size to 1/S2 using convolution and pooling operations, thereby reducing the computational cost and storage space of the feature maps. In our experiments, the number of heads is set to 8, with each head having a dimension of [64, 768]. While keeping the input and output matrices unchanged, MLP^31^ uses Dropout, DropPath, and Layer Norm to obtain suitable classification data, thereby reducing the risk of overfitting. Unlike the Linear and tanh activation function used by Ranftl ^17^, we employ the Linear and GELU^35^ activation function for data transformation to be compatible with the fine-tuning dataset. It is worth noting that the presence of the MLP block is not mandatory and does not impact average pooling or depth estimation.

The above steps can be summarized by the following four formulas:

1 $$ z_{l} = MLP\left( {LN\left( {z_{l}^{\prime } } \right)} \right) + z_{l}^{\prime } ,l = 1...L $$

where $z_{l}$ is the output of the current encoder block and $z^{\prime}_{l}$ is the output of the multi-head attention mechanism, respectively. following the input feature map has been linearly normalized, the dim is adjusted to quadruple and supplied to the GELU^35^ activation function to recover the categorical data. The matrix is then reset to [197,768] following random deactivation and another round of linear normalizing, $LN$ is Layer Normalize, which normalizes the input feature map.

2 $$ z_{l}^{\prime } = SRA\left( {LN\left( {z_{l - 1} } \right)} \right) + z_{l - 1} ,1...L $$

where $z_{l - 1}$ is the output of the previous Transformer encoder block and SRA is a multi-headed attention mechanism.

3 $$ z_{0} = \left\{ {X_{class} ;X_{P}^{1} E,X_{P}^{2} E;...X_{P}^{N} E} \right\} + X_{pos} ,E \in {\mathbb{R}}^{{\left( {P^{2} \cdot C} \right) \times D}} ,E_{pos} \in {\mathbb{R}}^{{\left( {N + 1} \right) \times D}} $$

where $X_{class}$ is the trainable label, $X_{P}^{N} E$ is N 2D Patches with resolution $P \times P$ for either 196 or 576, E is the trainable projection, and $E_{pos}$ is the positional embedding.

4 $$ y = LN\left( {z_{L}^{0} } \right) $$

where $z_{L}^{0}$ is the learnable classification embedding, $LN$ is Layer Normalize, which normalizes the input feature map, and $y$ is the classification result.

### Decoder

Following positional embedding, we incorporated the classical residual convolution module from ResNet^24^ to facilitate the fusion of markers with varying resolutions. Then, at each integration stage, we conducted multiple upsampling operations incrementally to achieve a final resolution of 1/S relative to the original resolution. Different S-fold values are employed for depth estimation and semantic segmentation, respectively.

It is noteworthy that within this process, we opted for layer normalization^31^ instead of the conventional batch normalization utilized in traditional ResNet methodologies. Our research upholds the principle of consistency, thereby enhancing the model's stability when employing PVT^29^ as the encoder during training.

We use the convolution operation to map the token from $N_{p}$ + 1 to $N_{p}$, by discarding the (class) token that contains the classification information.

5 $$ Mapping:{\mathbb{R}}^{{\left( {N_{P} + 1} \right) \times D}} \to {\mathbb{R}}^{{N_{P} \times D}} $$

According to the position of the initial patch, it is put into the corresponding position respectively to get the corresponding feature expression.

6 $$ Concatenate:{\mathbb{R}}^{{N_{P} \times D}} \to {\mathbb{R}}^{{\frac{H}{P} \times \frac{W}{P} \times D}} $$

A 1 × 1 convolution is used to change the channel, followed by a 3 × 3 convolution to resize.

7 $$ {\text{Re}} sample_{s} :{\mathbb{R}}^{{\frac{H}{P} \times \frac{W}{P} \times D}} \to {\mathbb{R}}^{{\frac{H}{S} \times \frac{W}{S} \times \hat{D}}} $$

S denotes the token that is assembled into a feature map with the spatial resolution of the $\frac{1}{S}$ input image.

In the reorganization and fusion phase of the feature map, we draw on the approach in^17^, while the formula can be expressed as:

8 $$ {\text{Re}} assemble_{s}^{{\hat{D}}} \left( t \right) = \left( {\left( {{\text{Re}} sample_{s} } \right) \circ \left( {Concatenate} \right)} \right)\left( t \right) $$

Finally, the feature map is upsampled and the decoding process is completed to obtain the depth map.

We use several methods such as [2,5,8,11], [3,6,9,12] for SPT-base and [5,11,17,23] for SPT-large.

## Model training

### Loss function

Loss is a crucial parameter to characterize the performance of the models in depth estimation. We employed a number of loss functions in the model development to minimize experimental errors brought on by human or non-human causes.

The loss function must quantify the “incorrectness”, because the depth map is more “continuous” than “discrete”. Mean Squared Error (MSE) is utilized as the loss function to calculate the loss of the depth map. SSI^22^ Truncation Function is used to provide a smooth depth estimation.

9 $$ {\mathcal{L}}_{MSE} = \frac{1}{n}\sum\limits_{i = 1}^{n} {\left( {y_{i} - \hat{y}_{i} } \right)}^{2} $$

where $y_{i}$ is the predicted value,$\hat{y}_{i}$ is the known target value of the depth map, and MSE is the mean value of the sum of squares of the differences between the two.

SSI loss can be viewed as a variant of MSE loss, or can be referred to as Shift and Scale Invariant Mean Square Error; they are essentially the same, for SSI loss:

10 $$ D\left( {y,y^{ * } } \right) = \frac{1}{2n}\sum\limits_{i = 1}^{n} {\left( {\log y_{i} - \log y_{i}^{ * } + \alpha \left( {y,y^{ * } } \right)} \right)}^{2} $$

where $y$ and $y^{ * }$ are the image data and the predicted data now, which refers to the actual depth value of each pixel and the predicted depth value. $\alpha$ is calculated as follows:

11 $$ \alpha \left( {y,y^{ * } } \right) = \frac{1}{n}\sum\limits_{i = 1}^{n} {\left( {\log y_{i}^{ * } - \log y_{i} } \right)} $$

SSI loss typically comprises two components. Equation (10) of the Scale Invariant Loss Term ensures that the model is insensitive to changes in scale. Usually, normalization or standardization operations are applied to both the predicted and true values to eliminate the influence of scale. Equation (11) of the Truncation Loss Term restricts the predicted values within a reasonable range by truncating them, thus mitigating the impact of outliers. Truncation is commonly achieved through trimming operations.

Since segmentation graphs are more "discrete" than “continuous", the loss function needs to be classified rather than quantified. For the segmentation objective, ‘Cross entropy^36^’ is used as the loss function for semantic segmentation.

12 $$ H_{p} \left( q \right) = \sum\nolimits_{x} {q\left( x \right)\log_{2} \left( {\frac{1}{p\left( x \right)}} \right)} \; = - \sum\nolimits_{x} {q\left( x \right)\log_{2} \left( x \right)} $$

where $p\left( x \right)$ is the output of the neural network, $q\left( x \right)$ is the correct solution label, and only the index of the correct solution label in $q\left( x \right)$ is 1 (may be other values), the rest is 0, so the equation x only calculates the natural logarithm of the output of the correct solution label. Each index corresponds to a $q\left( x \right)$ and an $H_{p} \left( q \right)$.

We employed "supervised training" to lower the cost of labeled training materials, alter the weights, and assess the depth map loss. In using SSI loss, we discard the gradient matching link and instead associate it with semantic segmentation. The total loss function^37^ proposed in this paper:

13 $$ {\mathcal{L}}_{com} = \alpha {\mathcal{L}}_{seg} + \beta {\mathcal{L}}_{depth} $$

where $\alpha$,$\beta$ are the impact factors, with values of $\alpha$ = 0.5,$\beta$ = 0.5.

In the joint loss function, depth estimation and semantic segmentation have equal weights. For semantic segmentation, the loss can be divided into two parts: cross-entropy loss and segmentation penalty term. The segmentation penalty term is the product of the difference between the current epoch's segmentation loss and the lowest segmentation loss, and the penalty factor (loss_seg_penality_factor). In the early stages of training, the penalty term can effectively suppress over-segmentation and under-segmentation.

As training progresses, the segmentation loss gradually decreases. At this point, the penalty term can make the model pay more attention to the details of the segmentation areas, thereby improving segmentation accuracy. The purpose of this is to improve certain aspects of depth estimation performance, such as enhancing the accuracy of edge depth estimation and reducing the impact of occlusion.

${\mathcal{L}}_{depth}$ incorporates three loss terms to achieve more accurate and high-quality depth maps by comprehensively considering various error sources. Specifically, SSI loss^22^ corrects the global scale of the predicted depth map to match the ground truth, the Smoothing loss suppresses noise and promotes smoothness, and the Structural similarity (SSIM)^38^ loss preserves structural details by measuring the structural similarity between the predicted and ground truth depth maps.

#### Smoothing loss

Smoothing loss is an optional component designed to help mitigate noise in the depth map. The specific formula is as follows:

14 $$ {\mathcal{L}}_{smooth} = \frac{1}{N}(\sum\limits_{i = 1}^{N - 1} {\sum\limits_{j = 1}^{M} {\left| {disp[i,j] - disp[i,j + 1]} \right|} + } \sum\limits_{i = 1}^{N} {\sum\limits_{j = 1}^{M - 1} {\left| {disp[i,j] - disp[i + 1,j]} \right|} } ) \times e^{ - \nabla I(x)} \times e^{ - \nabla I(y)} $$

where, $disp$ represents the predicted disparity image, while $i$ and $j$ denote the pixel position indices in the image. $N$ and $M$ correspond to the height and width of the disparity image, respectively. $- \nabla I(x)$ and $- \nabla I(y)$ signify the gradients of the color image in the horizontal and vertical directions, respectively. This loss function integrates the smoothness of the disparity image with the gradient information from the color image, aiming to enhance the model's sensitivity to edge variations while mitigating its sensitivity to scale and offset.

#### Structural similarity loss

SSIM loss^38^ is a crucial metric for assessing the accuracy of the reconstructed image. When compared with the mean square error function, SSIM can be used to compute the gray value of the corresponding pixel points in the original and reconstructed images, calculate the difference between them, and also structurally determine similarity of the two images . The formula is expressed as:

15 $$ {\mathcal{L}}_{SSIM} = \left( {x,y} \right) = \frac{{2\mu_{x} 2\mu_{y} + C_{1} }}{{\mu_{x}^{2} + \mu_{y}^{2} + C_{1} }} \times \frac{{2\delta_{xy} + C_{2} }}{{\delta_{x}^{2} + \delta_{y}^{2} + C_{2} }} $$

where $\mu_{x}$ and $\mu_{y}$ are the mean values of pixels of image $x$, $y$, $\sigma_{x}$ and $\sigma_{y}$ are the standard deviation of pixels of image $x$, $y$, $\sigma_{xy}$ is the pixel covariance of image $x$, $y$,$C_{1}$ and $C_{2}$ are set to avoid the denominator to be 0. A low SSIM loss means that the gap between the original and reconstructed images is small. The two reconstructed images are exactly the same, as the original image SSIM loss is 0.

### Coarse convergence and fine convergence

The model training is refined into two stages, i.e., coarse convergence and fine convergence.

#### Coarse convergence

In the coarse convergence stage (epochs < 10), convergence tends to be fast and non-smooth. For the final convergence, two aspects should be ensured.

1. The weights applied to the loss_in and loss_out formulas (16) must be normalized. SSI loss formulas (10) and (11) are used for this purpose.
2. For the desired segmentation to be accurate and noiseless, sufficient weights must be provided for the loss_Segmentation.
  16 $$ {\mathcal{L}}_{depth} = \gamma {\mathcal{L}}_{in} + \delta {\mathcal{L}}_{out} $$

“out" is the core of the study for depth estimation tasks, where “out” is the image loss that characterizes the depth > 0(foreground) part and “in” is the image loss that characterizes the depth < 0(background) part. Depth > 0 and depth < 0 will be treated separately because iteration will something produce reversal result. Something obvious depth > 0 will be interpreted as depth < 0 and vice versa. Therefore, we need to highlight the foreground(> 0) and background(< 0).

During the training process, we establish several dynamic hyperparameters that play a constructive role. We anticipate that these hyperparameters will dynamically influence the entire convergence process, enhancing the convergence speed and appropriately improving the model's adaptability at different training stages. As the number of epochs increases, these hyperparameters will gradually decrease and eventually stabilize.

Fx represents the global influence factor acting on the MSE loss^22^. Initially, its value is set to 2.5 at the beginning of training, but it gradually decreases as the training epoch progresses. If fx becomes too small, it introduces significant noise, thereby impeding the efficiency of model training. Therefore, to maintain training stability, the minimum threshold value for the global influence factor fx is set to 0.5. Additionally, when considering losses related to depth reduction, the minimum threshold value for fx is adjusted to 0.3 to ensure the preservation of finer depth details. Tab. 2 provides the values of these dynamic hyperparameters and their corresponding changes in loss.

The loss_coarse_threshold_factor is a factor specific to the coarse convergence stage (epochs < 10), which affects the overall joint loss function. Its initial value is set to 5. As training epochs progress, the impact of segmentation loss gradually becomes prominent, but the finer details of the predicted depth map remain relatively stable. When the loss_coarse_threshold_factor drops below 2.5, the finer details of the predicted depth map start to deteriorate, and when the factor value is 0, all details are lost. To prevent this scenario, we set its minimum threshold value to 2.5 as a safeguard.

It's worth noting that all loss parameters employed during the coarse convergence phase must facilitate precise and stable back-propagation. To address the challenge of lacking a loss function and associated parameters for covariance-invariant loss estimates in absolute depth, a scenario where depth estimates are expected to be perfectly accurate, a distinction is made between the validation loss and the training loss.

In cases where training and validation samples lack covariance invariance, loss estimates alone are insufficient to support stable and meaningful convergence. Therefore, when predicted values closely align with actual values, indicating a lack of covariance invariance in training and validation samples, SSI loss is introduced. This method ensures that the mean and standard deviation values across various source datasets are consistent, eliminating the need for a sample covariance-invariant processing step. Additionally, it's worth mentioning that the alpha value plays a role in gradient loss, with higher alpha values intensifying the impact of gradient loss on the overall loss. Experimental data indicate that the alpha value eventually stabilizes at 0.5.

#### Fine convergence

After the coarse convergence phase, the model training proceeds into the fine convergence stage (epochs > 10). During the coarse convergence stage, the influence of covariance invariance remains significant, allowing us to disregard the impact of fine convergence loss on the overall loss. In contrast, during the fine convergence phase, we introduce the concept of loss_fine_threshold_factor, which acts upon the joint loss function. Larger values of this factor result in more detailed depth maps. However, excessively large values of loss_fine_threshold_factor may lead to a reduction in the weight of segmentation loss, resulting in excessively noisy depth maps. Experimental results demonstrate that the optimal value for loss_fine_threshold_factor eventually stabilizes at 0.5.

As training epochs increase, determining the correctness of added details in the depth map becomes challenging. Correctly added details enhance the quality of the depth map, while incorrect additions diminish its quality. Unfortunately, there is no foolproof method at this stage to entirely eliminate incorrect details. The initial value for the fine convergence influence factor is set to 2.5, and as training progresses, the number of epochs increases while the influence factor gradually decreases. When adjusting fx_min from 0.5 to 0.7, other parameters are simultaneously tuned, resulting in improved accuracy of SPT-Depth compared to the ViT series models.

It's worth noting that ensuring the loss function of the segmentation map monotonically decreases throughout the entire training process is a challenging task. Any attempt to enforce strict monotonicity may introduce excessive damping, causing convergence to stop in fewer than 20 epochs. In this study, instead of strictly pursuing monotonicity, a penalty formula named loss_seg_penalty^37^ is proposed for the segmentation loss. This formula helps mitigate the bias of monotonically decreasing segmentation loss. The loss_seg_penalty_factor introduces controlled damping on convergence and must be carefully adjusted to avoid excessive values.

### Hyperparameterization

Our tests were performed using an Ubuntu 22.04 system with an Intel(R) Xeon(R) Silver 4210R CPU at 2.40 GHz, 8 × 32 GB DDR4 and 8 × TITAN XP with 12 GB of RAM. An NVIDIA TESLA V100 GPU graphics card was used to train the model. Python 3.10 and Pytorch 2.0.0 were used to implement the code. We pre-trained the model on INRIA Person Dataset (INRIA), NYU Depth V2 and Posetrack datasets. We compared the training loss and validation loss of SPT-Depth, fine-tuned ViT model^15^, with different parameter settings to obtain the optimal model results. Patch size is set to 16 or 32, respectively, while the training resolution was 384. Dropout is set to be 0.1 to avoid overfitting and improve model accuracy. Batch size can be 1 or 4, depending on the size of the model, the optimizer is Adaptive Moment Estimation (Adam)^39^ or Stochastic Gradient Descent (SGD)^40^, and the learning rate is uniformly 1e-5 for the backbone phase and 3e-4 for the coding and multi-head phases.

17 $$ w_{t + 1} = w_{t} - \mu \frac{1}{n}\sum\limits_{x \in \beta } {\nabla I\left( {x,w_{t} } \right)} $$

In this context, $n$ represents the batch size, $x$ is input, $\mu$ represents the learning rate, $w_{t}$ denotes the model parameter vector after the t-th iteration, and $\nabla I\left( {x,w_{t} } \right)$ signifies the gradient of the loss function $I$ with respect to the parameter $w_{t}$. It is evident that, in addition to gradients, these two variables directly influence the weight updates of the model, a critical parameter that significantly impacts the model's convergence. The convergence state and generalization performance of the model are intricately linked to both the numerator and denominator, with each being directly affected by the learning rate and batch size, respectively.An excessively high learning rate can prevent the model from converging, while an overly low learning rate can lead to very slow convergence. When the batch size is excessively large, the model may struggle to continue converging as it quickly approaches a local optimum, impeding its ability to reach the global optimum. Additionally, beyond a certain threshold, an excessively large batch size can adversely affect the model's generalization ability.Typically, below this threshold, changes in batch size have a relatively smaller impact on model performance compared to variations in the learning rate.

Table 1 presents a summary of the hyperparameter choices made in our experiments. These choices include dataset allocation, embedding dimension, model information extraction layers, optimizer selection, various loss functions for depth and segmentation, and the duration of training. To ensure the effective organization of the training dataset, we introduce the parameter "num_samples." Its primary purpose is to prevent excessive parameter tuning from causing the model to diverge. As "num_samples" increases, both the number of images and batches used in training also increase. The table provides an overview of these hyperparameter settings, excluding additional details related to hyperparameter configuration, such as Wandb data visualization, pathways, graph transformations, and so forth.

Table 2shows the key hyperparameters and training results that affect model convergence and stability. Among them, depth_datum acts as a scaling factor responsible for adjusting the output depth map to an appropriate range. The loss_seg_penality_factor serves as a segmentation penalty factor, penalizing the model during backpropagation by multiplying the difference between the segmentation loss of each epoch and the current lowest segmentation loss by this factor according to hyperparameters, thereby encouraging the model to minimize segmentation loss. During inference, the combined loss function prioritizes depth estimation accuracy by assigning it a 50% weight, while the remaining 50% is equally distributed between the cross-entropy loss and a segmentation penalty term. This configuration ensures that the model prioritizes accurate depth prediction while maintaining consistent segmentation boundaries. We incorporate MSE, SSIM, and Smooth as loss components with respective weights of 0.2, 0.5, and 0.5 to guide the optimization direction of the model. Notably, the global influence factor, fx, acts on the MSE loss component. For instance, when fx equals 2.5, the weight of the MSE loss is 0.5, gradually decaying to 0.1 after 10 epochs. This design aims to initially let the optimization process be predominantly governed by MSE; however, as MSE exerts its influence sufficiently, we gradually decrease its weight to match SSIM, ensuring that SSIM and Smooth can exert sufficient influence on the loss and further reduce the overall loss. The loss_ratio_out_factor serves as a weight attenuation factor for the output feature map, with higher values indicating a greater focus on the quality of the output feature map. Similarly, the loss_ratio_out_attenuation_factor represents the attenuation coefficient for the output attention map, with higher values resulting in smoother attention maps.

**Table 1:** Hyperparameters of SPT-Depth.

Variable Name	Explanation	Possible values
emb_dim	Dimension of the embeddings generated by the decoder	768 for base
		1024 for large
hooks	Refers to the layers that will be hooked	[2,5,8,11] ,[3,6,9,12] for base
		[5,11,17,23] for large
resample_dim	Refers to the decoder embeddings dimension	256
optim	Optmizer to use	SGD or Adam
lr_backbone	Learning rate to use for the backbone	Any float > 0 and < 1; Recommended: (with Adam) Since we use pre-trained weights1e-5
lr_scratch	Learning rate to use for the decoder and the multi-head module	Any float > 0 and < 1; Recommended: (with Adam)3e-4
loss_depth	Loss function to use for training the depth module	ssi for SSI loss
loss_segmentation	Loss function to use for the segmentation module	ce for CrossEntropy loss
epochs	Number of epochs for training	Any integer > 0 20, 50…
batch_size	Batch_size for training	Any integer > 0 we use 1, 4, 10…
patch_size	Different patch_sizes correspond to different SPT variants	16 for SPT-base
		32 for SPT-large
image_size	Our processing specifications for the images in the dataset	Uniformly organized in 384 × 384 format, with occasional use of 224 × 224 image sizes
num_samples	Used to prevent direct divergence due to too large a parameter	20, 40, 80, 160, 320, 640

**Table 2:** Hyperparameters and Training Outcomes.

	Scheme 1	Scheme 2	Scheme 3	Scheme 4	Scheme 5	Scheme 6	Scheme 7	Scheme 8
variable name								
depth_datum"	0.7	0.7	0.7	0.7	0.7	0.7	0.7	0.7
loss_seg_penality_factor"	3.0	3.0	3.0	3.0	3.0	3.0	3.0	3.0
loss_ratio_out_factor"	0.1	0.1	0.1	0.1	0.1	0.1	0.1	0.1
loss_mse_factor"	0.2	0.2	0.2	0.2	0.2	0.2	0.2	0.2
loss_ssim_factor"	0.5	0.5	0.5	0.5	0.5	0.5	0.5	0.5
loss_smooth_factor"	0.5	0.5	0.5	0.5	0.5	0.5	0.5	0.5
loss_ratio_out_attenuation_factor"	6.0	6.0	6.0	6.0	6.0	6.0	6.0	6.0
loss_segmentation_factor"	0.5	0.5	0.5	0.5	0.5	0.5	0.5	0.5
loss_depth_in_factor"	0.5	0.6	0.5	0.6	0.5	0.6	0.5	0.6
loss_depth_out_factor"	0.5	0.4	0.5	0.4	0.5	0.4	0.5	0.4
loss_fine_threshold_factor"	0.4	0.4	0.5	0.5	0.5	0.4	0.5	0.5
loss_coarse_threshod_factor"	5.0	5.0	5.0	7.0	5.0	5.0	5.0	5.0
fx	2.5	2.5	2.5	2.5	3.5	3.5	2.5	2.5
fx_min	0.5	0.3	0.5	0.5	0.5	0.3	0.5	0.5
Loss Type (epoch = 10)								
loss_seg_penality	0.0742	0.1314	0.1006	0.2111	0.1071	0.1021	0.1512	0.1500
loss_smoothness	0.0038	0.0035	0.0032	0.0084	0.0031	0.0039	0.0032	0.0031
loss_ssim	0.0262	0.0149	0.0120	0.0285	0.0151	0.0162	0.0148	0.0150
loss_mse	0.0757	0.0440	0.0885	0.2643	0.1467	0.1300	0.0771	0.0791
total loss	0.1800	0.1937	0.2043	0.5123	0.2720	0.2522	0.2463	0.2472
Loss Type (epoch = 45)								
loss_seg_penality	0.0002	0.0012	0.0007	0.0009	0.0005	0.0019	**0.0002**	0.0003
loss_smoothness	0.0033	0.0034	0.0034	0.0037	0.0037	0.0032	**0.0031**	0.0032
loss_ssim	0.0180	0.0203	0.0159	0.0213	0.0224	0.0215	0.0130	**0.0126**
loss_mse	0.0504	0.0576	0.0578	0.0630	0.0651	0.0643	**0.0361**	0.0374
total loss	0.0718	0.0825	0.0778	0.0889	0.0918	0.0909	**0.0524**	0.0535
accuracy	0.980	0.975	0.983	0.941	0.984	0.979	**0.996**	0.995

Significant values are in [bold].

In the latter part of the table, the experiment compared the parameter values and losses when the epoch was set to 10 and 45. Based on this, we can draw the following conclusions:

1. During the coarse convergence stage (epoch < 10), the segmentation loss based on segmentation penalty and MSE loss (SSI loss) dominate the optimization direction of the model, jointly controlling its optimization.
2. In the fine convergence stage, most losses have decreased to near their minimum values. At this point, the weight of the SSI loss decreases, and the loss values become close to SSIM and Smooth. All three jointly govern the continued optimization of the model.
3. Comparing Scheme1 and Scheme2, we conclude that further reducing fx_min indirectly increases the weight ratio of segmentation loss, aiding in preserving edge detail information, but without significant feedback on accuracy.
4. Comparing Scheme3 and Scheme4, we find that increasing the weight of the coarse convergence factor results in larger fluctuations in initial training losses. However, excessively low weights may lead the model to easily fall into local optima, hence this value should not be lower than 5.0.
5. Comparing Scheme5 and Scheme6, we conclude that the fine convergence factor significantly influences the values of segmentation loss and depth estimation loss. Decreasing this value further increases the weight of segmentation loss, leading to excessive noise, which is undesirable. Therefore, this value should not be lower than 0.5. Additionally, the value of fx is increased to 3.5, meaning that the weight of the SSI loss term is further increased during the coarse convergence stage. At this stage, the model primarily learns coarser image features, thus having minimal impact on the final outcome.
6. The final two sets of experiments adopt optimal parameter values, with differences only in weight values on depth > 0 and depth < 0. This measure enhances model robustness without causing drastic fluctuations in model loss and accuracy.

### Datasets

In depth estimation, we utilized three datasets: NYU Depth V2^41^ (640 × 480), INRIA^42^ (960 × 540), and Posetrack^43^ (1280 × 720) for model pre-training, as shown in Table 3. The evaluation of model accuracy was performed exclusively on the NYU Depth V2 dataset. Feng et al. previously utilized all available samples and employed them repeatedly in each epoch. However, this approach led to the reuse of all epoch samples, resulting in overfitting. In order to mitigate overfitting, we adopted a different strategy. Specifically, a predetermined number of samples, denoted as num_samples, were randomly selected during each epoch and subsequently shuffled randomly during training. To achieve higher accuracy, the value of num_samples was incrementally raised from an initial value of 20 to 160. It's important to note that using an excessively large number of samples would prolong the training time without yielding significant accuracy improvements.

**Table 3:** Loss comparison of different models.

Method	Epoch	Training loss	Validation loss
ViT-32	10	1.79	2.85
	20	1.45	2.59
	30	1.29	2.41
	45	**1.20**	**2.29**
ViT-16	10	1.55	2.62
	20	1.31	2.36
	30	1.20	2.25
	45	**1.14**	**2.20**
Hybrid	10	1.27	2.34
	20	1.17	2.21
	30	1.14	2.16
	45	**1.07**	**2.12**
PVT	10	1.21	2.31
	20	1.12	2.16
	30	1.10	2.12
	45	**1.06**	**2.08**
SPT-Depth	10	1.14	2.16
	20	1.08	2.07
	30	1.06	2.04
	45	**1.04**	**2.00**

Significant values are in [bold].

Supervised training necessitates a depth map with known true depth to validate and assess the loss. The NYU Depth V2 dataset contains ground truth depth estimation maps^44^, while the INRIA and Posetrack datasets serve to train the DPT-Hybrid model on images and generate depth maps as ground truth representations of the images ^17^.

For training, we allocated 60% of the dataset, while 20% each was reserved for validation and testing purposes. The depth map format is jpg, and despite being a grayscale image, the file format remains in 3-color format, denoted as (h, w, 3).

### Evaluation metrics

**Figure 5:**
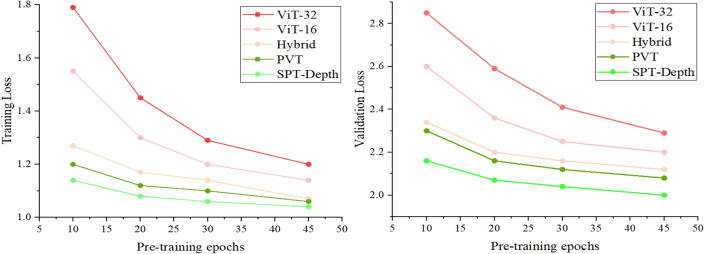
*Left*: Training loss between methods. During the fine-tuning phase, our method exhibits a convergence speed slightly lower than the baseline method (ViT), comparable to the PVT and Hybrid methods, reaching the lowest training loss at the 45th epoch of training. *Right:* Validation loss between methods. The validation loss decreases concurrently with the training loss, effectively preventing overfitting. The entire training process exhibits a smooth curve, demonstrating high stability.

The model training was conducted on the INRIA, NYU Depth V2, and Posetrack datasets. Pretraining lasted for 45 epochs. We compared the training and validation losses of the ViT-32 model ^15^, ViT-16 model ^15^, Hybrid model ^17^, PVT model ^29^, and our approach under different parameter settings to achieve the best model performance. The patch size was set to 16 or 32, and the training resolution was 384 × 384. A dropout of 0.1 was employed to prevent overfitting and improve model accuracy. Depending on the model's size, a batch size of 4 was used, and the optimizer selected was SGD^40^. The learning rate was set to 1e-5 during the backbone stage and 3e−4 during the decoder stage. We trained different methods for 10, 20, 30, and 45 epochs to observe convergence behavior. Table 3 provides a comparison of the training and validation losses for different models, and Fig. 5 offers a visual representation of the data from Table 3.

In the experiments, larger models exhibited lower accuracy due to limitations in the dataset. However, as the dataset size increased, the accuracy gradually improved. The research findings highlight the significant advantage of our approach over other models in terms of training and validation losses. (1) Compared to other methods, SPT-Depth adopts a lower learning rate during the fine convergence phase, resulting in a relatively slower convergence speed. However, SPT-Depth ultimately achieves lower losses and higher robustness, indicating its stronger fitting and generalization capabilities. (2) Our method incorporates various loss functions, including Smoothing loss, SSIM loss, and SSI loss, which significantly outperform the baseline (ViT). (3) We define the entire training process as consisting of two phases: fine convergence and coarse convergence. We employ different training strategies and hyperparameters, resulting in lower training and validation losses starting from the fine convergence stage. Further fine-tuning adjustments, including parameters like loss_fine_threshold_factor, contribute to improved training accuracy. (4) The training process should not exceed 45 epochs, as training on a small dataset can lead to overfitting.

The depth estimate results in this study are assessed using the depth estimation assessment metrics^45^, which take into account both accuracy and error, with less errors being preferable to bigger accuracy values. The precise experimental quantitative assessment metrics are described as follows:

18 $$ RMSE = \sqrt {\frac{1}{\left| N \right|}\sum\limits_{i \in N} {\left| {\left| {d_{i} - d_{i}^{ * } } \right|} \right|}^{2} } $$

19 $$ Abs{\text{Re}} l = \frac{1}{\left| N \right|}\sum\limits_{i \in N} {\frac{{\left| {d_{i} - d_{i}^{ * } } \right|}}{{d_{i}^{ * } }}} $$

20 $$ Accuracy = \% of\;di\;s.t.\max \left( {\frac{{d_{i} }}{{d_{i}^{ * } }},\frac{{d_{i}^{ * } }}{{d_{i} }}} \right) = \delta < thr $$

where $d_{i}$ is the predicted depth value of pixel $i$ and $d_{i}^{ * }$ is the actual depth value,$N$ is the total number of pixels with actual values, $thr$ is the threshold value. The threshold accuracy is to calculate the ratio of the predicted depth to the actual depth of all pixels in the image, take the maximum value, and finally assign the result to $\delta$. The ratio of the pixels whose $\delta$ is less than the threshold $thr$ to the total pixels is the correctness accuracy. The closer the result to 1, the better the result is. $thr$ is generally taken as 1.25, ${1.25}^{2}$ and ${1.25}^{3}$.

All models in Table 4, were retrained using the INRIA, NYU Depth V2, and Posetrack datasets, and validation was conducted on NYU Depth V2. The selected models are all designed for depth estimation tasks, with the majority having undergone fine-tuning on NYU Depth V2. We have endeavored to maintain consistency in training strategies and hyperparameter fine-tuning, building upon this foundation to ensure experimental fairness.

**Table 4:** Performance comparison on V2 dataset.

Method	Lower is better		Higher is better			Lower is better
	Abs Rel	RMSE	${\updelta }_{1}$	${\updelta }_{2}$	${\updelta }_{3}$	Params
ViT-B/16 2020^15^	0.115	0.440	0.901	0.985	0.992	1.1G
ViT-L/16 2020^15^	0.120	0.551	0.875	0.977	0.981	1.2G
DPT-Hybrid 2021^17^	0.110	0.357	**0.904**	0.988	0.988	473M
AdaBins 2021^46^	0.112	0.409	0.891	0.965	0.986	919M
SABV 2023^19^	**0.101**	0.421	0.894	0.977	0.989	873M
Chen 2022^47^	0.135	0.828	0.828	0.963	0.981	1.1G
Ozay 2021^48^	0.118	0.571	0.857	0.957	0.982	1.6G
Runze 2022^49^	0.132	0.517	0.834	0.961	0.990	1.6G
Lite-mono 2023^20^	0.121	0.520	0.871	0.940	0.981	**3.1M**
Lite-mono-8M 2023^20^	0.125	0.541	0.867	0.925	0.979	8.0M
LapDepth 2021^50^	0.112	0.445	0.875	0.956	0.971	282M
Ours	0.109	**0.351**	0.895	**0.988**	**0.996**	151M

Significant values are in [bold].

The bolded part is the best. It is found that both the absolute relative error and root mean square error clearly favor SPT-Depth in terms of accuracy. At the corresponding threshold values of ${\updelta }_{1}$ < 1.25, ${{\updelta }_{2} < 1.25}^{2}$, ${{\updelta }_{3} < 1.25}^{3}$, the model accuracy is on par with that of the other models. In comparison to the state-of-the-art method, the RMSE score has decreased by 17% (0.421 vs. 0.351), with only a slight increase in the Abs Rel metric (0.101 vs. 0.109). Given that lightweighting is not the focus of the experiment, SPT-Depth has a larger parameter scale compared to Lite-mono and Lite-mono-8M. However, in comparison to methods of similar types, SPT-Depth has a smaller parameter scale, reducing by 86% relative to the baseline (ViT).

**Figure 6:**
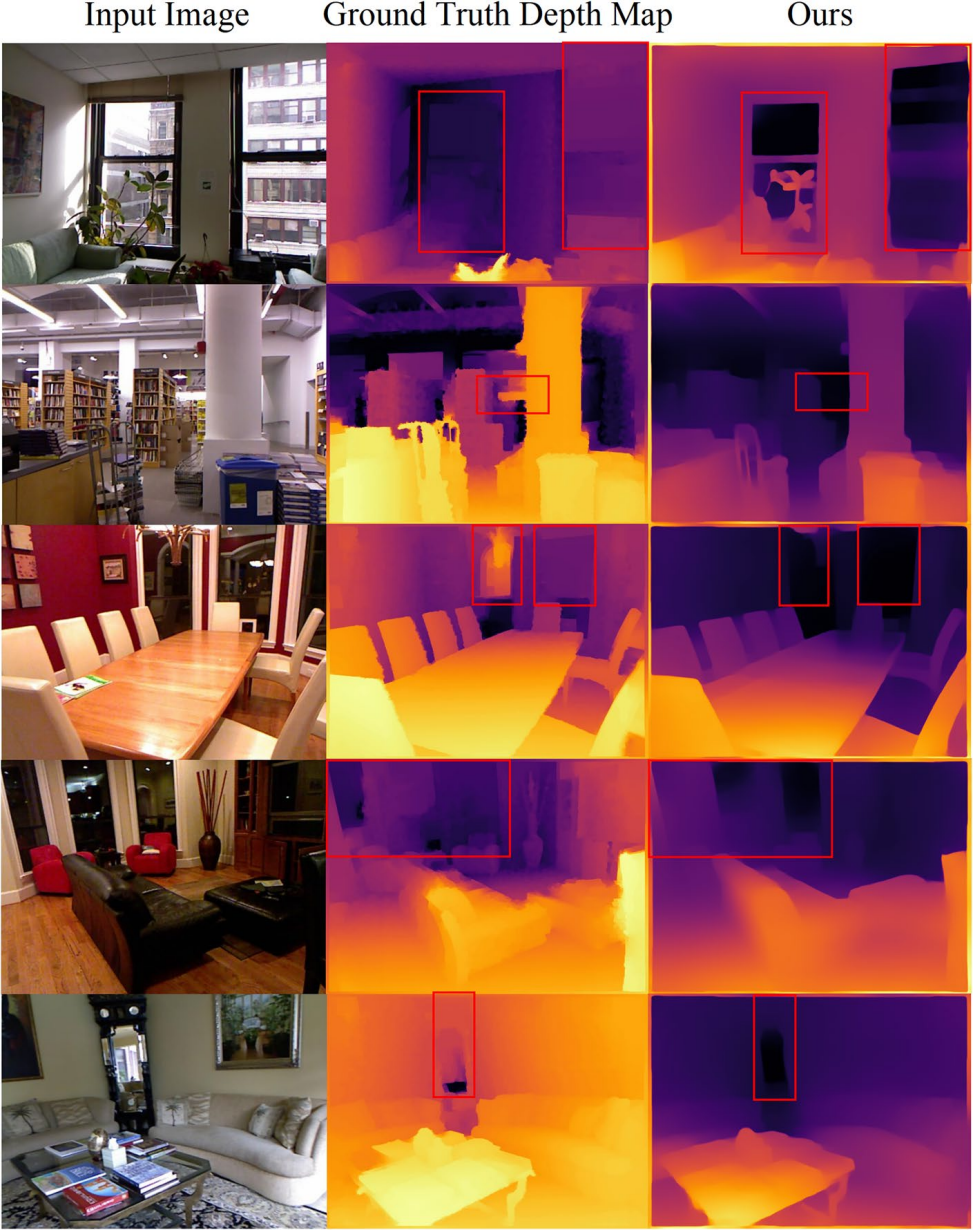
Comparison with the Ground Truth Visualization. In the NYU Depth V2 dataset, certain portions of the ground truth depth maps exhibit some discrepancies. For instance, in the first image, windows and flowers are not accurately identified, and in the second image, the window in the farthest distance is mistakenly assigned a lower depth value. It is noteworthy that our approach, following training on the NYU Depth V2, INRIA, and Posetrack datasets, shows a degree of resilience in mitigating the aforementioned issues.

We have observed that the depth maps in the NYU Depth V2 dataset contain relatively small errors and incomplete image information. If the model is trained solely on the NYU Depth V2 dataset, these errors and incompleteness would persist. Therefore, we adopt a training approach that utilizes not only the NYU Depth V2 dataset but also the INRIA and Posetrack datasets. As shown in Fig. 6, the encouraging results of subsequent tests on the NYU Depth V2 dataset indicate the model's significant generalization capability. It partially compensates for errors in real ground information, although this change may not be accurately reflected in terms of precision.

**Figure 7:**
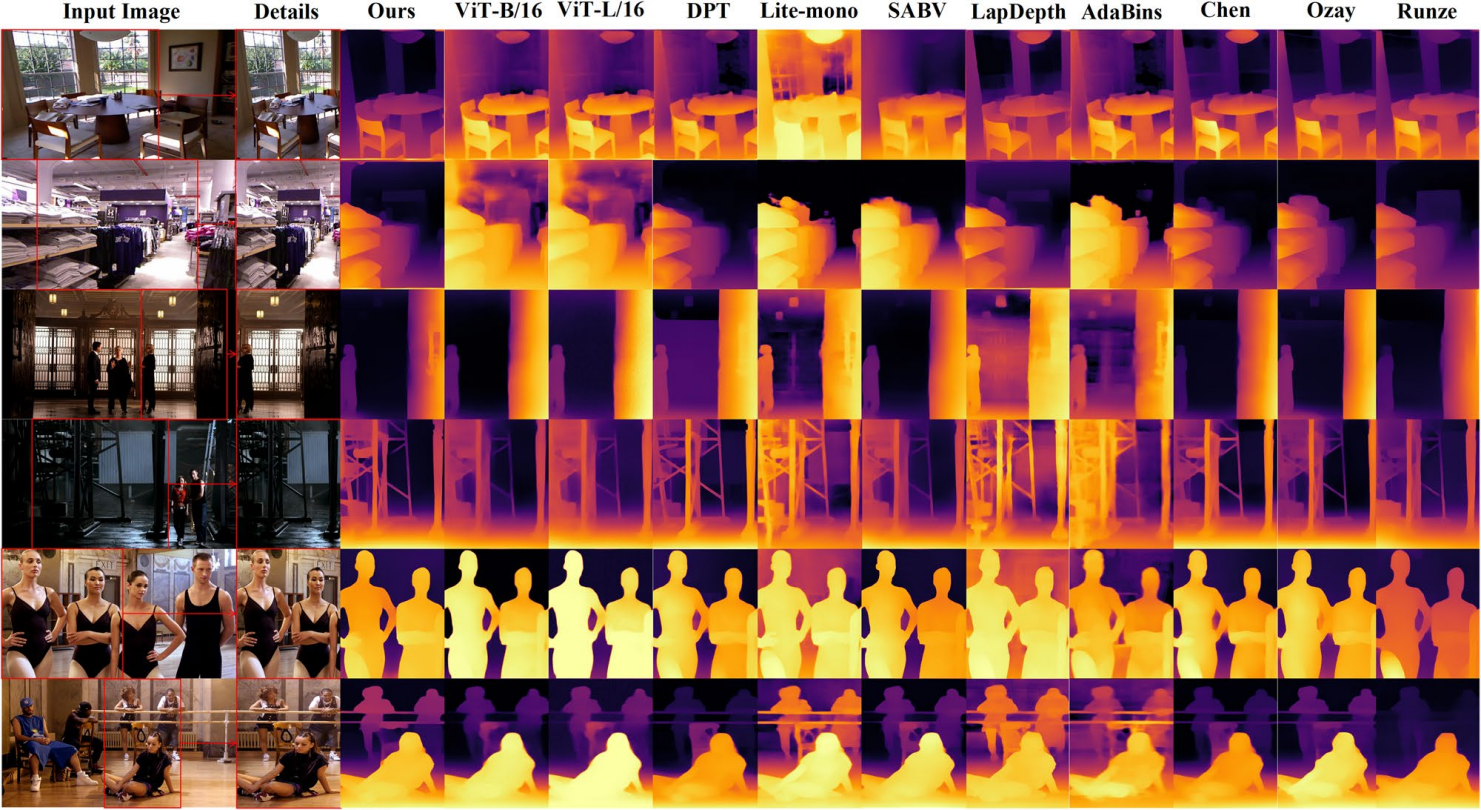
Visualization Comparison. As presented in Table 4. The six sets of images are arranged in top-to-bottom order, sourced respectively from the NYU Depth V2, INRIA, and Posetrack datasets (with every two sets originating from the same dataset).

Figure 7 compares the visualizations of all methods in Table 4 on the NYU Depth V2, INRIA, and Posetrack datasets. We specifically zoom in on details of some images, especially indoor scenes and portraits, to highlight the superior performance of our method. For example, in the first image, our method retains object edge information more completely; the fourth image excels in recognizing fine details in outdoor scenes; and the sixth image identifies parallel railings more accurately. While SPT-Depth's estimation may lack in certain details, this is a result of the training strategy. In simple terms, if we cannot accurately estimate the depth values of certain farther parts, we uniformly set their depth values to 0 (in visualization). For instance, in the first image, Lite-mono^20^ identifies more details, but it incorrectly estimates the depth information of these details, resulting in the depth value of the windows behind being similar to the foreground furniture, which is clearly not what depth estimation aims to achieve. Similar situations also occur in the third image of LapDepth (Laplacian Depth Estimation Network)^50^ and AdaBins (Adaptive Bins Transformer-Based Depth Estimation Network)^46^, where the images identify chandeliers but cannot accurately estimate their depth, resulting in a chaotic representation in the image.

**Figure 8:**
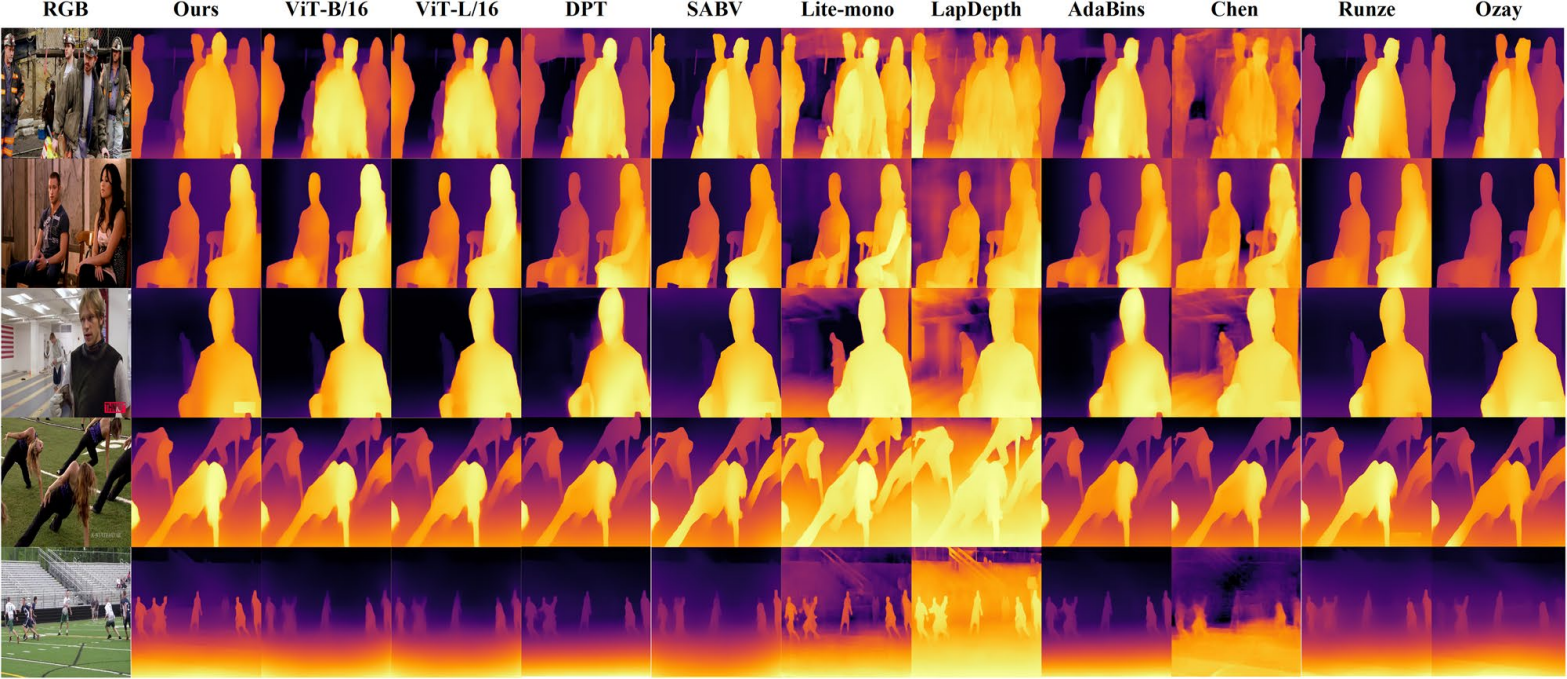
Visualization Comparison on Posetrack Dataset. The images contain multiple individuals simultaneously, with significant variations in their depth information, thus better reflecting the performance of the models in depth estimation.

Figure 8 presents the visual results of the methods in Table 4 on the Posetrack dataset. We selected images with more people to further demonstrate the outstanding performance of our method in estimating human depth. For instance, the depth prediction of two people in the second image is more accurate; the third image prominently retains the text details in the lower right corner; and the fifth image demonstrates excellent edge prediction capabilities while accurately identifying the depth information of multiple individuals in the scene. Similar to Fig. 7, in the fifth image, both Lite-mono and LapDepth identify more details behind the people, but these details are incorrect, as evidenced by their depth values being similar to the foreground individuals and exhibiting inversion (the colors behind are lighter, indicating higher depth values); in the first image of the method proposed by Chen et al., more detailed image information is displayed, but similarly, it fails to accurately predict the depth values of these details (depth values are close, and depth values are set to 0 in unreasonable locations).

**Figure 9:**
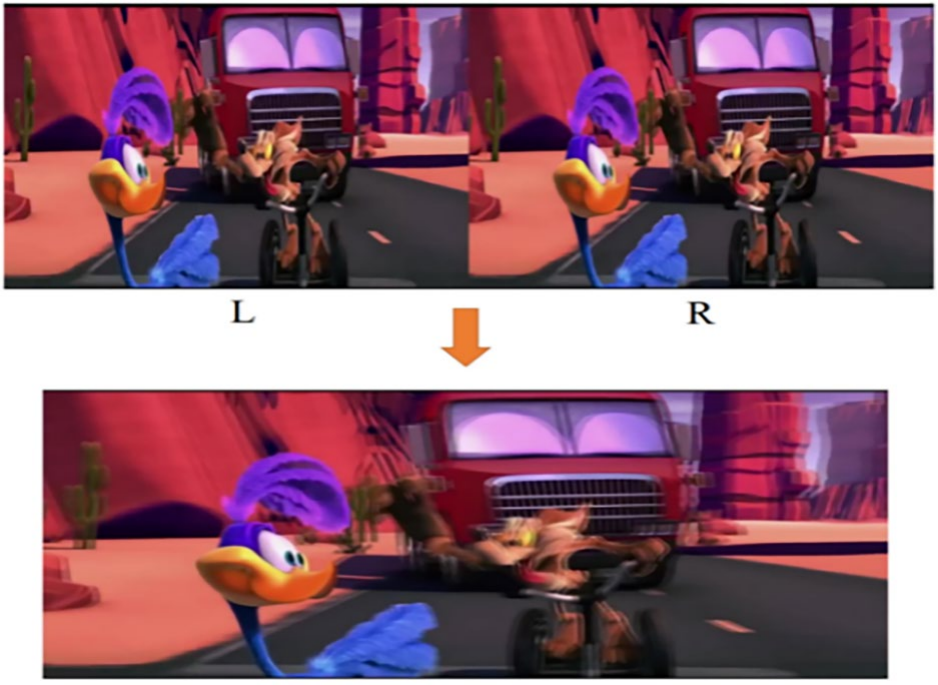
Left and right eyes see different images.

The multi-view image reconstruction is depicted in Fig. 9 The image was captured by horizontally shifting the camera by 65 mm, which corresponds to the average human inter-pupillary distance, in front of the screen. The two images were fused to simulate the perspectives seen by the left and right eyes, respectively^32^. When observing the screen, subtle distinctions exist between the images perceived by the left and right eyes, providing our brains with a perception of three-dimensionality.

### Ablation experiment

We conducted an ablation study using the NYU Depth V2 dataset and provided the quantified results of the PVT backbone, Linear SRA, and SSI loss as summarized in Table 5. Initially, we used the ViT-large model as a baseline. Subsequently, we replaced the ViT backbone with the PVT backbone while retaining the MHA, leading to the second set of experiments. The third set of experiments, based on the second set, replaced the MHA with Linear SRA. Finally, the fourth set of experiments, building on the third set, replaced the MSE loss function with SSI loss.

**Table 5:** Ablation study on V2 dataset.

Components			NYU depth V2	
PVT backbone	Linear SRA	SSI loss	Abs rel	RMSE
			0.120	0.551
√			0.115	0.461
√	√		0.114	0.459
√	√	√	**0.109**	**0.351**

Significant values are in [bold].

In Table 5, we use bold font to indicate results that achieved the best performance. When we replaced the ViT-based backbone with the PVT-based backbone, we observed a 16% decrease in the RMSE metric. This suggests that the PVT backbone can better learn long-range contextual information, thereby improving matching accuracy. Furthermore, when Linear SRA replaced the MHA module, the changes in the Abs Rel and RMSE metrics were not significant, indicating that Linear SRA has a relatively minor impact on model performance. Finally, with the introduction of SSI loss, Abs Rel decreased to 0.109, and RMSE decreased to 0.351. This indicates that SSI loss indeed contributes to improving the model's performance, bringing the model to an optimal state.

## Conclusions

Depth estimation plays a crucial role in achieving autostereoscopic displays. We have constructed a dense depth estimation network by introducing the Pyramid Transformer as the backbone, leveraging its advantages in dense prediction tasks. We reduced the dimension of feature maps through downsampling to decrease the number of tokens required, thus reducing computational demands. Simultaneously, by employing embedding, upsampling, reassemble, and a series of convolution operations, we restored multi-scale feature maps to the same resolution size and fused shallow and deep information to output high-precision depth maps.

The introduction of the SSI loss successfully addresses the issue of the lack of translational invariance in Transformers. The structural similarity function and edge-smoothing function reduce noise in the depth map, enhancing the model's robustness. The model's pretraining process is divided into two stages, coarse convergence and fine convergence, employing different training strategies, parameters, and hyperparameters, leading to a significant reduction in both training and validation losses.

Experiments were conducted on the INRIA, NYU Depth V2, and Posetrack datasets. The results indicate that, compared to other methods, our approach exhibits lower training loss. The network architecture and training methods of SPT-Depth effectively improve the accuracy of depth prediction while preventing overfitting.

However, the model has some limitations. For example, in certain regions of the image where clear and blurred sections converge, artifacts may appear, potentially affecting 3D display quality. Additionally, while our approach reduces computational demands compared to ViT-based methods, the computational complexity issue has not been completely resolved.

SPT-Depth effectively captures object boundaries and contours, aiding in understanding semantic information within the scene and providing solutions to the aforementioned issues. We plan to bridge the semantic gap between the encoder and decoder using a self-supervised monocular depth network framework, starting from the encoder-decoder intermediate. To further improve rendering results and reduce artifacts, we can incorporate classic computer vision algorithms or end-to-end techniques. To address computational complexity comprehensively, we plan to introduce strategies from the Swim Transformer to confine attention calculations within each window, thereby significantly reducing computational demands.

## Data Availability

Dense Monocular Depth Estimation for Stereoscopic Vision Based on Pyramid Transformer and Multi-Scale Feature Fusion by Zhongyi Xia is licensed under CC BY 4.0 All images containing people used in this paper are from the publicly available datasets inria and posetrack and do not involve human experimentation. All personally identifiable information/images used in this article are sourced from publicly available datasets, namely, INRIA and PoseTrack. The relevant statements have already been included in "Alahari, K., et al. Pose Estimation and Segmentation of People in 3D Movies. in 2013 IEEE International Conference on Computer Vision. 2013." and "Andriluka, M., et al. PoseTrack: A Benchmark for Human Pose Estimation and Tracking. in 2018 IEEE/CVF Conference on Computer Vision and Pattern Recognition. 2018." The data that support the findings of this study are available from the corresponding author upon reasonable request. Posetrack Dataset Dataset website: https://paperswithcode.com/dataset/posetrack Licenses: https://creativecommons.org/licenses/by-nc/4.0/Paper: https://ieeexplore.ieee.org/document/8578640 Inria movie 3d Dataset Dataset website: https://www.di.ens.fr/willow/research/stereoseg/Licenses: https://creativecommons.org/licenses/by-sa/4.0/ Paper: https://ieeexplore.ieee.org/document/6751373/metrics#metrics.

## References

[1] Miangoleh, S. M. H., Dille, S., Mai, L., Paris, S. & Aksoy, Y. Boosting monocular depth estimation models to high-resolution via content-adaptive multi-resolution merging. *Proc. 2021 IEEE/CVF Conference on Computer Vision and Pattern Recognition (CVPR)*, 9680–9689 (2021).

[2] Zhou, H., Greenwood, D., Taylor, S. L. & Gong, H. Constant velocity constraints for self-supervised monocular depth estimation. *Proc. of the 17th ACM SIGGRAPH European Conference on Visual Media Production* (2020).

[3] Krizhevsky A, Sutskever I, Hinton GE (2012). ImageNet classification with deep convolutional neural networks. Commun. ACM.

[4] Shelhamer, E., Long, J. & Darrell, T. Fully convolutional networks for semantic segmentation. *Proc. 2015 IEEE Conference on Computer Vision and Pattern Recognition (CVPR)*, 3431–3440 (2014). 10.1109/TPAMI.2016.2572683 27244717

[5] Noh, H., Hong, S. & Han, B. Learning deconvolution network for semantic segmentation. *Proc. 2015 IEEE International Conference on Computer Vision (ICCV)*, 1520–1528 (2015).

[6] Ronneberger, O., Fischer, P. & Brox, T. U-Net: Convolutional networks for biomedical image segmentation. *ArXiv***abs/1505.04597** (2015).

[7] Godard, C., Mac Aodha, O. & Brostow, G. J. Digging into self-supervised monocular depth estimation. *ArXiv***abs/1806.01260** (2018).

[8] Charles Leek E, Leonardis A, Heinke D (2022). Deep neural networks and image classification in biological vision. Vis. Res..

[9] Zhao Z, Yang H, Luo H (2022). Defocus Blur detection via transformer encoder and edge guidance. Appl. Intell..

[10] Zhao C, Dai M, Xiong J-Y (2016). Region-of-interest based rate control for UAV video coding. Optoelectron. Lett..

[11] Huang, H. *et al.* UNet 3+: A full-scale connected UNet for medical image segmentation. *Proc.**ICASSP 2020 - 2020 IEEE International Conference on Acoustics, Speech and Signal Processing (ICASSP)*, 1055–1059 (2020). 10.1109/ICASSP40776.2020.9053555 PMC7543994 33041676

[12] Eigen, D., Puhrsch, C. & Fergus, R. in *Neural Information Processing Systems.*

[13] Vaswani, A. *et al.* in *Neural Information Processing Systems.*

[14] Liu, M., Meng, F. & Liang, Y. Generalized pose decoupled network for unsupervised 3D skeleton sequence-based action representation learning. 10.34133/cbsystems.0002 PMC10076048 37040281

[15] Dosovitskiy, A. *et al.* An image is worth 16x16 words: Transformers for image recognition at scale. *ArXiv***abs/2010.11929** (2020).

[16] Li, Z., Liu, X., Creighton, F. X., Taylor, R. H. & Unberath, M. Revisiting stereo depth estimation from a sequence-to-sequence perspective with transformers. *Proc.**2021 IEEE/CVF International Conference on Computer Vision (ICCV)*, 6177–6186 (2020).

[17] Ranftl, R., Bochkovskiy, A. & Koltun, V. Vision transformers for dense prediction. *Proc.**2021 IEEE/CVF International Conference on Computer Vision (ICCV)*, 12159–12168 (2021).

[18] Liu, Z. *et al.* Swin Transformer: Hierarchical vision transformer using shifted windows. *Proc.**2021 IEEE/CVF International Conference on Computer Vision (ICCV)*, 9992–10002 (2021).

[19] Wang J (2023). SABV-depth: A biologically inspired deep learning network for monocular depth estimation. Knowl. Based Syst..

[20] Zhang, N., Nex, F., Vosselman, G. & Kerle, N. Lite-mono: A lightweight CNN and transformer architecture for self-supervised monocular depth estimation. *Proc.**2023 IEEE/CVF Conference on Computer Vision and Pattern Recognition (CVPR)*, 18537–18546 (2022).

[21] Yang, L. *et al.* Depth Anything: Unleashing the power of large-scale unlabeled data. *ArXiv***abs/2401.10891** (2024).

[22] Brébisson, A. d. & Vincent, P. The Z-loss: A shift and scale invariant classification loss belonging to the Spherical Family. *ArXiv***abs/1604.08859** (2016).

[23] Mayer, N. *et al.* A large dataset to train convolutional networks for disparity, optical flow, and scene flow estimation. *Proc.**2016 IEEE Conference on Computer Vision and Pattern Recognition (CVPR)*, 4040–4048 (2015).

[24] He, K., Zhang, X., Ren, S. & Sun, J. Deep residual learning for image recognition. *Proc.**2016 IEEE Conference on Computer Vision and Pattern Recognition (CVPR)*, 770–778 (2015).

[25] Lyu, X. *et al.* HR-depth: High resolution self-supervised monocular depth estimation. *ArXiv***abs/2012.07356** (2020).

[26] Peng, R., Wang, R., Lai, Y., Tang, L. & Cai, Y. Excavating the potential capacity of self-supervised monocular depth estimation. *Proc.**2021 IEEE/CVF International Conference on Computer Vision (ICCV)*, 15540–15549 (2021).

[27] Carion, N. *et al.* End-to-end object detection with transformers. *ArXiv***abs/2005.12872** (2020).

[28] Rao, Y., Zhao, W., Zhu, Z., Lu, J. & Zhou, J. Global filter networks for image classification. *ArXiv***abs/2107.00645** (2021).

[29] Wang, W. *et al.* Pyramid vision transformer: A versatile backbone for dense prediction without convolutions. *Proc.**2021 IEEE/CVF International Conference on Computer Vision (ICCV)*, 548–558 (2021).

[30] Zheng Q, Yu T, Wang F (2023). Self-supervised monocular depth estimation based on combining convolution and multilayer perceptron. Eng. Appl. Artif. Intell..

[31] Tolstikhin, I. O. *et al.* in *Neural Information Processing Systems.*

[32] Li Y, Claesen L, Huang K, Zhao M (2019). A real-time high-quality complete system for depth image-based rendering on FPGA. IEEE Trans. Circuits Syst. Video Technol..

[33] Zhou Y, Zhang J, Fang F (2022). Design of the varifocal and multifocal optical near-eye see-through display. Optik.

[34] Wang W (2021). PVT v2: improved baselines with pyramid vision transformer. Comput. Vis. Med..

[35] Shaw, P., Uszkoreit, J. & Vaswani, A. in *North American Chapter of the Association for Computational Linguistics.*

[36] Rajamani KT, Rani P, Siebert H, Elagiri Ramalingam R, Heinrich MP (2023). Attention-augmented U-Net (AA-U-Net) for semantic segmentation. Signal Image Video Process..

[37] Mousavian, A., Pirsiavash, H. & Kosecka, J. Joint Semantic Segmentation and Depth Estimation with Deep Convolutional Networks. *Proc.**2016 Fourth International Conference on 3D Vision (3DV)*, 611–619 (2016).

[38] Cao, Y., Luo, F. & Li, Y. in Image and Graphics: 12th International Conference, ICIG 2023, Nanjing, China, September 22–24, 2023, Proceedings, Part I 81–92 (Springer-Verlag, Nanjing, China, 2023).

[39] Loshchilov, I. & Hutter, F. Fixing weight decay regularization in Adam. *ArXiv***abs/1711.05101** (2017).

[40] Horváth S, Kovalev D, Mishchenko K, Richtárik P, Stich SU (2022). Stochastic distributed learning with gradient quantization and double-variance reduction. Optim. Methods Softw..

[41] Silberman, N., Hoiem, D., Kohli, P. & Fergus, R. *Proc.**European Conference on Computer Vision.*

[42] Alahari, K., Seguin, G., Sivic, J. & Laptev, I. *Proc.**2013 IEEE International Conference on Computer Vision.* 2112–2119.

[43] Andriluka, M. *et al.**Proc.**2018 IEEE/CVF Conference on Computer Vision and Pattern Recognition.* 5167–5176.

[44] Bian J (2020). Auto-rectify network for unsupervised indoor depth estimation. IEEE Trans. Pattern Anal. Mach. Intell..

[45] Zhang, H. *et al.* ResNeSt: Split-attention networks. *Proc.**2022 IEEE/CVF Conference on Computer Vision and Pattern Recognition Workshops (CVPRW)*, 2735–2745 (2020).

[46] Bhat, S., Alhashim, I. & Wonka, P. AdaBins: Depth estimation using adaptive bins.* Proc.**2021 IEEE/CVF Conference on Computer Vision and Pattern Recognition (CVPR)*, 4008–4017 (2020).

[47] Chen Y, Zhao H, Hu Z (2019). Attention-based context aggregation network for monocular depth estimation. Int. J. Mach. Learn. Cybern..

[48] Hu, J., Ozay, M., Zhang, Y. & Okatani, T. Revisiting single image depth estimation: toward higher resolution maps with accurate object boundaries. *Proc.**2019 IEEE Winter Conference on Applications of Computer Vision (WACV)*, 1043–1051 (2018).

[49] Li R, Ji P, Xu Y, Bhanu B (2023). MonoIndoor++: Towards better practice of self-supervised monocular depth estimation for indoor environments. IEEE Trans. Circuits Syst. Video Technol..

[50] Song, M., Lim, S. & Kim, W. Monocular Depth Estimation Using Laplacian Pyramid-Based Depth Residuals. *IEEE Trans. Circuits Syst. Video Technol.* **31**, 4381–4393 (2021).

